# Reporting and evaluating genetic association studies

**DOI:** 10.1186/1465-9921-10-109

**Published:** 2009-11-12

**Authors:** Stephen P Peters

**Affiliations:** 1 Wake Forest University Health Sciences, Center for Human Genomics, Winston-Salem, NC, USA

## Abstract

Genetic association studies have become an important part of our scientific landscape. This commentary discusses some basic scientific issues which should be considered when reporting and evaluating such studies including SNP Discovery, Genotyping and Haplotype Analysis; Population Size, Matching of Cases and Controls, and Population Stratification; Phenotype Definition and Multiple Related Phenotypes; Multiple Testing; Replication; Genome-wide Association Studies (GWAS); and the Role of Functional Studies. All of these elements are important in evaluating such studies and should be carefully considered when these studies are conceived and carried out.

## Introduction

Genetic association studies have become an important part of our scientific landscape. They add a unique perspective to our understanding of the pathogenesis of disease, and often present unique candidates for mechanistic studies. The number of such studies has been increasing exponentially: a recent Pub Med search for "Genetic Association Studies" limited to "humans" and English" resulted in more than 24,000 citations. The form that these studies are taking is being driven not only by scientific issues (scientific questions, availability of well characterized populations), but also by rapid advances in technology. Whereas it was once reasonable to report one or a few single nucleotide polymorphisms (SNPs) in an association study, advances in our ability to perform high through-put DNA sequencing and genotyping mandate that current publications report a more comprehensive analysis of genetic variations in a target, than would have been required in the recent past. In fact, chips are now available which can identify more than 370,000 to as many as 1 million SNPs, which span the entire human genome. In part because of these capabilities, the NHLBI has recently funded 13 centers (STAMPEED) to perform genome-wide association studies in heart, lung, blood, and sleep disorders; 3 of these centers focus on asthma and lung diseases.

In view of such advances, what new metrics do the scientific community and this Journal require for articles submitted for publication? These issues are also of interest to other journals, as shown by the publication of perspectives which discuss these issues [1-3]. Accordingly, several aspects of study design are paramount when submitting a manuscript for publication. These include: 1) Adequacy of SNP discovery, completeness of the genotyping, and adequacy of haplotype analysis; 2) Study population size, the matching of cases and controls, and whether the issue population stratification has been addressed; 3) Definition of phenotype for primary and secondary analyses, and the examination of multiple, interrelated phenotypes; 4) How the issue of multiple testing and multiple comparisons has been addressed; 5) Replication of positive findings in additional study populations, if they are available, and the extent to which biologic plausibility can substitute for replication, particularly if replicate populations are unavailable; 6) Approach to genome-wide association studies; and 7) Role of functional studies in association studies. Table 1 lists these elements and our comments concerning them. A number of recent articles have addressed some or all of these issues [4-9]. Other reviews, which also include the topic of genome-wide association studies (GWAS), are also informative [10-12].

**Table 1:** Criteria for Evaluating Genetic Association Studies

Elements	Comment
**SNP Discovery and Genotyping (Completeness)**	SNP discovery and genotyping of a gene should be complete (based on current literature AND re-sequencing of the gene in a subgroup of the population studied).
	
**Haplotype Analysis**	Haplotype analyses should be reported.
	
**Population Size**	Size should be large enough to have reasonable power to avoid both false negative and false positive results
	
**Matching Cases and Controls**	Appropriate matching is always a consideration
	
**Population Stratification**	Population stratification should always be addressed (in some manner)
	
**Phenotype Definition**	The best genetic association studies employ a robust definition of the phenotype (i.e. a physician's diagnosis of asthma is much less robust than one based on physiologic and clinical criteria)
	
**Multiple Testing (Comparisons)** **Inter-Related Phenotypes**	Issue of multiple testing must always be addressed, while acknowledging that some phenotypes are inter-related (and not independent), and Bonferroni correction may be too conservative
	
**Replication and Role of Functional Studies**	Replication is necessary unless it cannot be reasonably performed. In some cases, functional studies substitute for replication

## SNP Discovery, Genotyping and Haplotype Analysis

While there are now many on-line resources which describe the variability (SNPs) in genes of interest in different populations (*e.g. *HapMap Project), these tools may be incomplete, not representative of the population studied, may not include important functional polymorphisms, and may be inaccurate. With recent advances in sequencing technologies, re-sequencing of target genes is much less onerous than in the past. For this reason, re-sequencing of the target gene(s) in either the target population, or a comparable one, should be considered to confirm/identify all important variations in the gene, and to devise an efficient genotyping strategy for the population under investigation. The number of individuals which should be sequenced depends on the whether only common SNPs are of interest, or whether rare variations in the gene are also of interest [13]. Currently, there is little justification to report incomplete genotyping for any genetic target in an association study.

Genotyping methods have evolved greatly over the past decade. Older approaches such as single-stranded conformational polymorphism (SSCP), denaturing high performance liquid chromatography (dHPLC), and amplified-refractory mutation system (ARMS), have largely given way to newer and more reliable techniques such as TaqMan, Sequenom (mass spectrometry), and rapid throughput sequencing. Some variants, such as SNPs in repetitive elements, can prove problematic for genotyping. Therefore investigators should confirm the accuracy of at least a subset of genotypes using a second technique to ensure accuracy. Results must be evaluated for Hardy-Weinberg equilibrium. Haplotype analyses should be routinely performed and reported. As we have become more efficient in these technologies reporting analyses of pathways containing multiple genes, rather than a single gene should be expected. There is little justification for reporting a single or few SNPs in a single gene.

## Population Size, Matching of Cases and Controls, and Population Stratification

Recent publications have stressed the importance of adequate population size, the matching of cases and controls, and the related issue of population stratification [1,3,5,9,14]. As recently stated by Hunter [3] citing Wacholder [15], "a "statistically significant" finding in an underpowered study is more likely to be a false positive result due to chance than is such a finding in an adequately powered study, and "statistically significant" associations could be attributed to systemic bias (*e.g. *from confounding due to ethnic ancestry, also known as population stratification.)" Hall [1] has recently published a table with a suggested number of cases according to genotype relative risk, minor allele frequency, and dominant/recessive effect. For example, for a genotype relative risk of 1.5, a dominant effect, and a minor allele frequency of 0.05, 1,300 cases would be required for an α of 0.05 and power of 90%. However, only several hundred cases (300 to 750) would be required for a larger genotype relative risk and/or a larger minor allele frequency. A recessive effect would require more cases than a dominant one. In addition, cases and controls need to be matched for important variables, including ethnic background. While this can often be done on the basis of shared geography, this approach is not always sufficient. The genotyping of ancestry informative markers (AIMs) to address population stratification is important, particularly as the cost of genotyping continues to fall. Another approach may use family-based association studies [*e.g. *[16]]. The large number of SNPs identified in GWAS studies provides another mechanism for performing this analysis.

## Phenotype Definition and Multiple Related Phenotypes

One of the most important considerations in genetic association studies concerns phenotype description, namely, 1) the phenotype definition; and 2) use of multiple, often inter-related phenotypes.

A phenotype under investigation can often be characterized using various definitions, which can differ in accuracy. For example, an "asthma" phenotype can be assumed based on patient provided answers to a questionnaire; because a patient has received a physician's diagnosis of "asthma;" or because a physician's diagnosis has been confirmed by physiological testing (*i.e. *bronchodilator reversibility and/or airway hyperresponsiveness to methacholine). The last definition is more stringent than the first two, and different phenotype definitions might lead to the inability to replicate a genetic association.

Some phenotypes can be defined using multiple definitions. For example, bronchodilator reversibility can be defined as the absolute change in FEV_1 _after bronchodilator administration, the percent change in FEV_1 _from baseline, or the change based on the FEV_1 _percent predicted. If an association for bronchodilator reversibility is "replicated" in two populations using different definitions for bronchodilator reversibility, should some statistical adjustment be made for these multiple comparisons/multiple definitions?

Finally, many genetic association studies explore several related phenotypes. For example, genetic associations studies in asthma often include the phenotypes "asthma" (defined by one or more ways described above; bronchial hyperresponsiveness; serum IgE level; lung function (often in several different ways such as FEV_1_, FEV_1_/FVC ratio, etc.); and bronchodilator reversibility. Rationales for how statistical adjustments are performed when examining multiple, highly correlated endpoint should be clearly stated.

Finally, while many genetic association studies are truly exploratory, the most robust studies test a single or limited number of specific hypotheses, based on biologic plausibility and/or previous findings. These considerations help formulate the basis of a sound statistical analysis plan that can be convincingly described for both reviewers and readers.

## Multiple Testing

All genetic association studies must address the issue of multiple testing and comparisons [1,3,9]. While it is widely considered that the Bonferroni correction is too conservative a correction for most purposes, the standard p value of 0.05 is similarly too liberal. Approaches to this problem include a formal permutation analysis [17]) or (somewhat arbitrarily) choosing a more stringent p value for significance (*e.g. *0.01 or 0.001). One can also choose the highest ranked SNPs in an exploratory population (from a threshold p value or other factor), to *identify a limited number of SNPs *to be examined in a replication population. Whether independent or interdependent phenotypic endpoints are examined raise another important consideration for multiple testing. That is, a finding might not be considered to be "replicated" if marginal p values (*e.g. *p = 0.04) were observed in two populations for the same SNP, but with different definitions of the same phenotype (*e.g. bronchodilator reversibility *defined as the absolute increase in FEV_1 _in one population and as the % increase over baseline in a second population, or *lung function *defined as FEV_1 _in one population and FEV_1_/FVC ratio in a second population).

## Replication

Any positive finding in a genetic association study needs to be replicated in one or more additional populations, assuming such population(s) exist [1,3,5,9,18]. This is particularly important in genome-wide association studies [2,3,6,10,12]. Exactly what constitutes replication, however, needs to be considered from the point of view of both of the SNP(s) involved and the phenotype(s) replicated.

In the simplest case, an association is replicated if the same SNP or haplotype is associated with the phenotype, *in the same direction*, in two or more populations. (In the recent past, some might have considered [and may still consider] an association replicated, even if the direction of the phenotypic association were in opposite directions in two populations.) A finding can also be considered to be replicated if SNP1 is associated with a phenotype in population 1 and SNP2 is associated with the same phenotype in population 2, *if SNP1 and SNP2 are in perfect linkage disequilibrium (D' or R^2 ^of 1.0)*. Two measures of linkage disequilibrium are in common usage, D' and r^2^. D' measures unidirectional linkage disequilibrium (*e.g. *knowing the value of SNP1, you are certain of the value of SNP2; however, knowing the value of SNP2, you may not know the value of SNP1). r^2 ^is a measure of bidirectional linkage disequilibrium (*e.g. *it is the traditional correlation of SNP1 and SNP2). Whether a D' or r^2 ^value less than 1.0 (such as 0.8) is sufficient to use SNP2 as a surrogate for SNP1 in a replication study is open to debate. Finally, in attempting to replicate a specific finding (SNP1) in a second population, investigators may fail to replicate the specific SNP (SNP1), but then discover stronger associations in additional closely related SNPs; this could occur if SNP1 were not in strong linkage disequilibrium with the causal SNP, while the newly associated SNPs were [9]. Approaches using systemic meta-analytic techniques have been suggested as another approach for replicating genetic association studies [18].

Should a finding be considered replicated if the same SNP/haplotype were found to be associated in two populations, not with exactly the same phenotype, but in a related one (*e.g. *different definitions of the same phenotype [asthma defined by doctor's diagnosis versus physiologic criteria) or through the use of two related phenotypes (*e.g. *lung function defined by FEV_1 _and FEV_1_/FVC ratio)? Such circumstances require interpretation by the investigators, reviewers and editors. Among the factors which should be considered when considering this issue are 1) how close either the different phenotype definitions or related phenotypes track with one another, and 2) the strength of the associations. Strong statistical associations (P < 0.001) with related phenotypes, are more convincing than weak associations (p = 0.04).

## Genome-wide Association Studies (GWAS)

Genome-wide association studies have both their own advantages, and concerns, recently discussed in a number of commentaries and articles [2,3,6,10,12]. All of the issues discussed above are also pertinent for genome-wide association studies, with an emphasis on the issue of replication. In this case, multiple populations are required, often using a technique of nested replication. A recent report provides useful information concerning power calculations for genome-wide association studies [19]. In addition, the effects of copy number variations (CNVs) should also be considered. Finally, "a central challenge in this area is the development of powerful multipoint methods that can detect variants that have not been directly genotyped" [20]. Accordingly, techniques have recently been reported for addressing the issue of missing data in genome-wide analyses using the techniques of imputation [20] and Markov chain haplotyping [21].

## Role of Functional Studies

Functional studies which can help clarify mechanism can counterbalance deficiencies in some genetic association studies. Such data, however, should not be required in all genetic association studies since SNPs found to be important in genetic association studies may produce no change in protein expressed (synonymous), may be located in non-coding regions (introns or 5' or 3' untranslated regions), and/or may require an intact organism/animal/human in order to demonstrate physiological relevance.

## Conclusion

This commentary discusses many of the issues that must be addressed in performing and reporting genetic association studies, including SNP discovery, genotyping and haplotype analysis; population size, matching of cases and controls, and population stratification; phenotype definition and the issue of multiple related phenotypes; multiple testing; replication; and the special issues of genomewide association studies and functional studies. All of these issues must be addressed to ensure high quality genetic association studies. The extent, to which authors can address these issues, plus the novelty and importance of their observations, will play a major role in determining the suitability of the manuscript for publication.

## Abbreviations

SNP(s): single nucleotide polymorphism(s); GWAS: genome-wide association studies; SSCP: single-stranded conformational polymorphism; dHPLC: denaturing high performance liquid chromatography; ARMS: amplified-refractory mutation system; AIMS: ancestry informative markers.

## Competing interests

None with the subject of this commentary, except that it was prepared, in part, in conjunction with my responsibilities as an Associate Editor of Respiratory Research, for this journal.
